# Supplementary material for: Petermann ice shelf may not recover after a future breakup
Source: Nat Commun. 2022 May 9;13:2519. doi: 10.1038/s41467-022-29529-5 (PMC9085824; doi:10.1038/s41467-022-29529-5)
Supplement: Supplementary file 1 — Supplementary Information [file 41467_2022_29529_MOESM1_ESM.pdf]

Supplementary Information for the article *Petermann Ice Shelf may not recover after a future breakup*

Henning Åkesson[1,2,3], Mathieu Morlighem[4,5], Johan Nilsson[2,6], Christian Stranne[1,2], and Martin Jakobsson[1,2]

[1]Department of Geological Sciences, Stockholm University, Sweden
[2]Bolin Centre for Climate Research, Stockholm University, Sweden
[3]Department of Geosciences, University of Oslo, Norway
[4]Department of Earth Sciences, Dartmouth College, Hanover, NH 03755, USA
[5]Department of Earth System Science, University of California, Irvine, CA, USA
[6]Department of Meteorological Sciences, Stockholm University, Stockholm, Sweden

Corresponding author email: henning.akesson@geo.uio.no

1 Overview

In this Supplement a few additional details are presented about calving seasonality (Section S1.1), basal friction (Section S1.2) and glacier mass balance (Section S1.3). Finally, an overview table of model experiments is provided (Table S1), as well as details about the data sources used (Section S1.5).

1.1 Calving seasonality

Recovery of Petermann relies on a change of the calving regime, in tandem with an ocean cooling (Section Reduced calving as an escape route from pervasive mass loss). This holds regardless of whether the calving regime is seasonal, or is represented as an annual average. In the main text, experiments using an annual calving forcing are described. We also performed simulations using a seasonal calving regime, with high calving rates (low stress threshold) in summer and low calving rates (high stress threshold) in winter (cf. Equation 2 in main text). The summer calving season is here defined as April to September, and winter is October to March (6 months each). At each seasonal transition, the stress threshold σ_{max} is changed stepwise, mimicking the onset and the end of the calving season [1].

Regardless of whether the calving forcing is annual or seasonal, a high enough calving stress threshold (>∼440 kPa; Fig. S8) needs to be imposed to allow calving-front advance to the present-day ice-shelf configuration. For recovery to succeed, this critical calving stress threshold however only needs to be exceeded during the winter, and can remain low (100–300 kPa) during the summer (Fig. S9kl and Table S1). This implies that if calving is reduced enough during winter, for example by a strong ice mélange (Section Sea-ice induced advance of ice shelves), this can trigger a readvance that may be sustained even under a vigorous summer-calving regime.

1.2 Basal friction

The bulk of simulations presented in the main text are based on a Budd-type friction law (Methods: Ice-flow model). We also tested whether our findings about ice-shelf recovery are robust with a Schoof friction law [2, 3]. Basal drag $\boldsymbol{\tau}_{b,S}$ using this friction law is calculated as

$$\boldsymbol{\tau}_{b,S} = -\frac{C_s|\mathbf{u}_b|^{m-1}\mathbf{u}_b}{\left(1+\left(\frac{C_s}{C_{max}N}\right)^{1/m}|\mathbf{u}_b|\right)^m} \tag{1}$$

where C_s is a friction parameter and $m = 1/n$, where $n = 3$ is the exponent in Glen's flow law.

The basal friction parameter C is found such that the Schoof law (Eq. 1) gives the same basal drag as does the Budd law (Eq. 1 in main text), that is $\boldsymbol{\tau_{b,S}} = \boldsymbol{\tau_{b,B}}$ [4, 5].

Solving for C_s in Eq. 1 gives:

$$C_s = \frac{|\boldsymbol{\tau}_{b,B}|}{|\mathbf{u}_b|^m \left(1 - \left(\frac{|\tau_{b,S}|}{C_{max}N}\right)^{1/m}\right)^m}. \tag{2}$$

We set $C_{max} = 0.6$ as spatially constant, and set C_s in ice-free and floating areas to an arbitrary low value of 10^{-3} MPa $m^{-1/3}$ $a^{1/3}$, following Brondex et al. [4] and Åkesson et al. [5]. The same value for C_s is used for localized grounded areas where $|\tau_{\mathbf{b},\mathbf{B}}| > C_{max}/N$, that is, where the so-called Iken's bound [6] is violated (see Brondex et al. [4] and Åkesson et al. [5] for details). At Petermann this amounts to localized areas across a few km from the grounding line.

Additional simulations using the Schoof law confirm that our findings about recovery remain robust (Section Study limitations and future work). Specifically, recovery still fails for an ocean cooling (Fig. S5 and Fig. S6) and for an 'ocean cooling + increased surface mass balance' (Table S1).

The simulated grounding-line discharge during [5] and after ice-shelf breakup is however 20–25% lower for a Schoof law than for the Budd friction law (Figure S7).

Recovery also fails both starting from a retreated state associated with a 2°C and a more extreme 5°C-warming (as simulated in Åkesson et al. [5]; Fig. S5 and S6). Clearly, a reduced calving needs to complement the ocean cooling to allow recovery of the ice shelf, regardless of the friction law chosen.

1.3 Glacier mass balance

In Figure S10 we detail the evolution of each component of Petermann's glacier mass balance for an extended set of model simulations.

The balance between mass gain Q_{in} and mass loss Q_{out} is calculated as

$$dV/dt = Q_{in} - Q_{out} = Q_{acc} - (Q_{abl} + Q_b + Q_c), \tag{3}$$

where Q_{acc} is surface accumulation, Q_{abl} is surface ablation, Q_b is basal melt, and Q_c is the calving flux. The degree of recovery is robust over multiple time scales, from instant (change over 1 year), fast (100 years), intermediate (250 years), to slow (500 years). For clarity, 'ocean-only' in Figure S10 means an ocean cooling; 'calving-only' means an increased calving stress threshold (σ_{max} in Eq. 2 in main text) and thus reduced calving; 'ocean + calving' means ocean cooling and reduced calving; 'ocean + SMB' means ocean cooling and increased surface mass balance. See main text for further details. For calving, we include simulations with a weak (summer/winter σ_{max}: 200/400 kPa; Fig. S10k), moderate (summer/winter σ_{max}: 150/450 kPa; Fig. S10l) and strong (summer/winter σ_{max}: 200/1000 kPa; Fig. S10m) seasonality. We also show simulations with an annual calving forcing ($\sigma_{max} = 500$ kPa; Fig. S10e–j). These experiments are all included in Table S1.

1.4 Experiment overview

Here we list all experiments mentioned in the text, with the associated model forcings.

1.5 Data information

The underlying data used here is the same as used in Åkesson et al. [5]. The data is all freely available and listed in Table S1.5. The data can be downloaded from the following sources:

- Bedrock topography, BedMachine v3 [8]: https://doi.org/10.5067/2CIX82HUV88Y
- Ice surface topography [9, 10]: https://doi.org/10.5067/2CIX82HUV88Y, contained within BedMachine v3 [8]
- Land mask [9, 10]: https://doi.org/10.5067/2CIX82HUV88Y, contained within BedMachine v3 [8]
- Ice velocities [11, 12]: https://doi.org/10.5067/QUA5Q9SVMSJG
- Grounding line position [13]: http://products.esa-icesheets-cci.org/products/details/greenland_grounding_line_locations_v1_3.zip/
- Calving front position [14]: http://products.esa-icesheets-cci.org/products/details/greenland_calving_front_locationsv3_0.zip
- Surface mass balance [7]: ftp://ftp.climato.be/fettweis/MARv3.5.2/Greenland/ERA-int_1979-2014_10km/monthly_outputs_interpolated_at_5km/MARv3.5.2-10km-yearly-ERA-Interim-nc
- Ocean-induced melt [15]: https://doi.org/10.1002/2017GL073711 (available upon request from the corresponding author)

2 Supplementary Figures

Table S 1: Overview of experiments performed in this study. For the ocean forcing, the temperature relative to the present-day is listed; associated melt rates are shown in Fig. S2. For the atmosphere, the positive shift in the surface mass balance (SMB) at sea level and summit is listed as 'sea level/summit' (m w.e. change relative to the present-day surface mass balance; Methods: Climate forcing; [7]). For calving, the tensile stress threshold σ_{max} (Eq. 2 in main text) is included. For experiments listed as 'Ocean cooling + reduced calving, σ_{max} sensitivity', the sensitivity to σ_{max} is tested (Fig. S8). In experiments with seasonal calving, the stress threshold is listed as the values for 'summer/winter' (Section 1.1). Otherwise, the annual σ_{max} is listed. For time scale, the number of years over which changes are imposed is listed. For example, a 100-year time scale means that the forcing is changed linearly over a 100-year period, and is kept constant afterwards, until the end of the simulation. Additional experiments using a Schoof friction law is also shown (Section S1.2).

**Experiment**	**Ocean (°C)**	**SMB (m w.e.)**	**Calving stress (kPa)**	**Time scale (years)**	**Recovery (yes/no)**
Core experiments					
Retreat, 2°C	+2	present	300	1	-
Ocean cooling	present	present	300	1	no
Ocean cooling + increased surface mass balance	present	+1/+0.2	300	1	no
Ocean cooling + reduced calving	present	present	500	1	yes
Additional experiments					
Control	+2	present	300	1	no
Calving-only	present	present	500	1	no
Calving-only, strong	present	present	1000	1	no
Ocean-off	no melt	present	300	1	no
Ocean cooling (fast)	present	present	300	100	no
Ocean cooling (intermediate)	present	present	300	250	no
Ocean cooling (slow)	present	present	300	500	no
Ocean cooling + reduced calving (fast)	present	present	500	100	yes
Ocean cooling + reduced calving (intermediate)	present	present	500	250	yes
Ocean cooling + reduced calving (slow)	present	present	500	500	yes
Ocean cooling + reduced calving, σ_{max} sensitivity	present	present	300–440	1	no
Ocean cooling + reduced calving, σ_{max} sensitivity	present	present	441–600	1	yes
Ocean cooling + reduced calving, weak seasonal	present	present	200/400	1	no
Ocean cooling + reduced calving, moderate seasonal	present	present	100/500	1	yes
Ocean cooling + reduced calving, strong seasonal	present	present	200/1000	1	yes
Ocean cooling + increased SMB, strong	present	+2/+0.2	300	1	no
Retreat, 5°C	+5	present	300	1	-
Retreat, 2°C, Schoof	+2	present	300	1	-
Retreat, 5°C, Schoof	+5	present	300	1	-
Ocean cooling, 5°C	present	present	300	1	no
Ocean cooling, 2°C, Schoof	present	present	300	1	no
Ocean cooling, 5°C, Schoof	present	present	300	1	no
Ocean cooling, 5°C + increased SMB	present	+1/+0.2	300	1	no
Ocean cooling, 2°C + increased SMB, Schoof	present	+1/+0.2	300	1	no
Ocean cooling, 5°C + increased SMB, Schoof	present	+1/+0.2	300	1	no
Ocean cooling, 5°C + reduced calving	present	present	500	1	yes
Ocean cooling, 2°C + reduced calving, Schoof	present	present	500	1	yes
Ocean cooling, 5°C + reduced calving, Schoof	present	present	500	1	yes

Table S 2: Overview of data sources used in this study.

Data set	Name	Time period	Reference
Bedrock topography	BedMachine v3	2017	[8]
Ice surface topography	GIMP v1	2003–2009	[16, 10]
Land mask	GIMP v1	2007–2008	[9, 10]
Ice velocities	MEaSUREs v1	1995–2015	[11, 12]
Grounding line position	ESA CCI GLL v1.3	2017	[13]
Calving front position	ESA CCI CFL v3.0	2008	[14]
Surface mass balance	MAR v3.5.2	1979–2014	[7]
Ocean-induced melt		2008	[15]

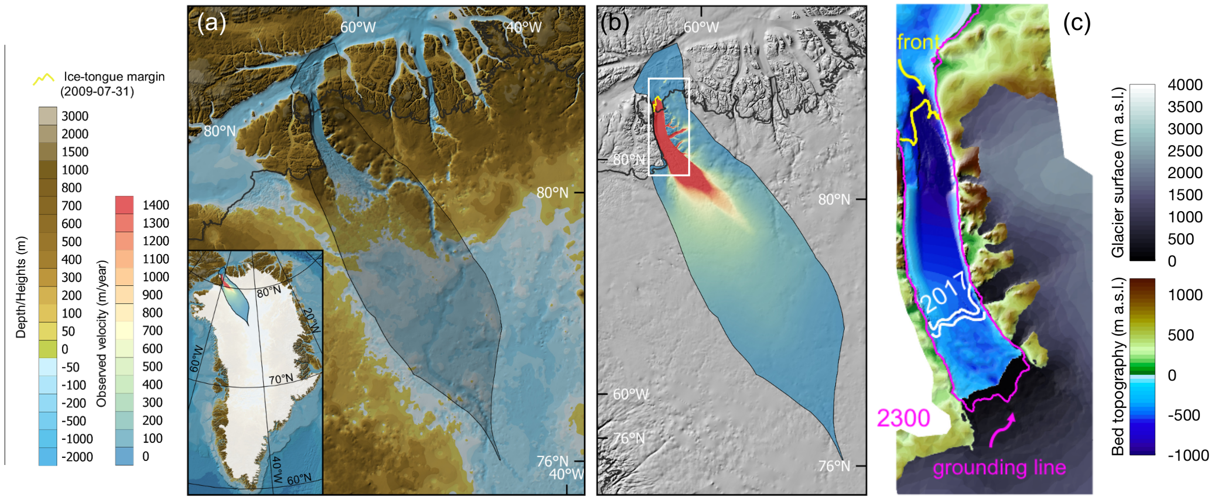

Figure S 1: (a) Petermann Glacier model domain and bed topography, with inset of the Greenland Ice Sheet, (b) observed ice velocities [11, 12], (c) geometry and grounding line at the end of the 'future' warming experiments, used as initial conditions in the 'recovery experiments'. In (c), the glacier state after a 2°C warming is shown [5]. The yellow line is the present-day front, the white lines represent the present-day grounding zone, and the magenta shows the modelled grounding line in year 2300 AD [5].

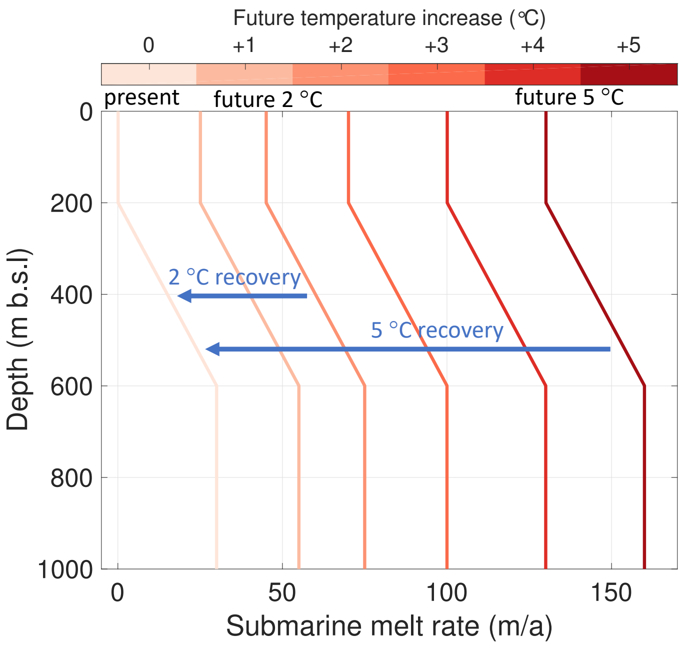

Figure S 2: Depth – melt rates profiles used in model simulations. Melt rates are based on MITgcm ocean model results from Cai et al. [15]. Shifts of the depth – melt rates curves for 'ocean-cooling experiments' are shown with arrows.

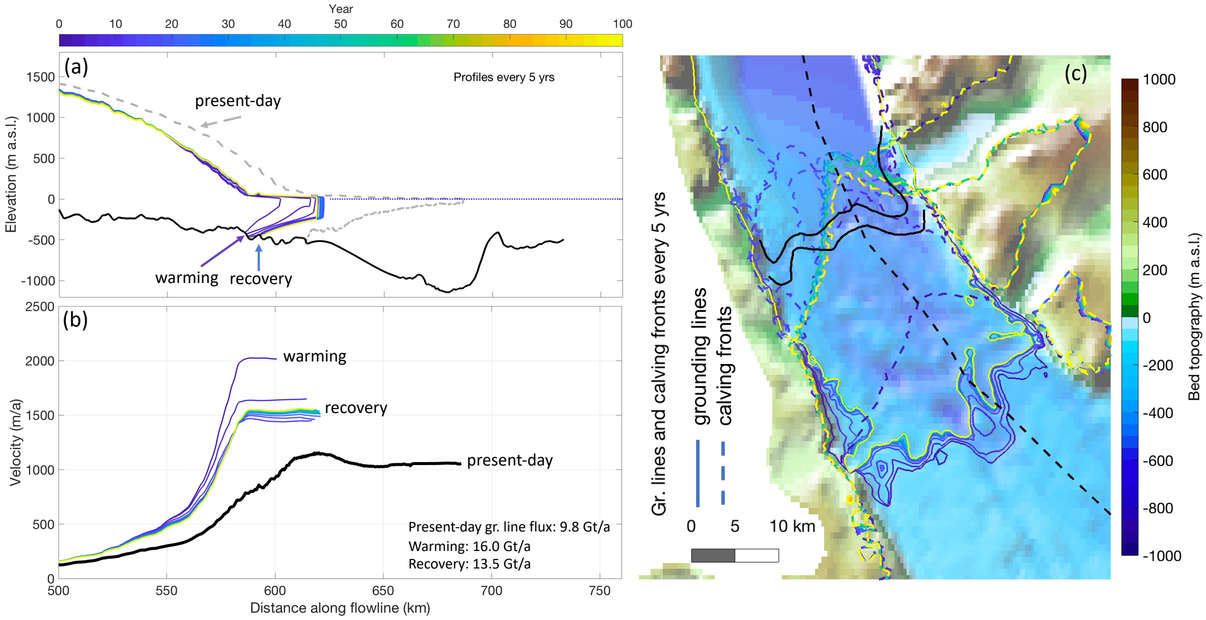

Figure S 3: Ocean-cooling recovery following a 2°C ocean warming. Only the first 100 years of the simulation are shown for visibility; negligible change occurs over the rest of the simulation. Ocean forcing is reversed back to present-day conditions, yet Petermann is unable to recover. Grounding-line flux after recovery remain 40% higher than at present. (a) Geometry evolution along the flowline shown as dashed black in (c); (b) velocity evolution along flowline; (c) planview grounding-line and calving-front evolution.

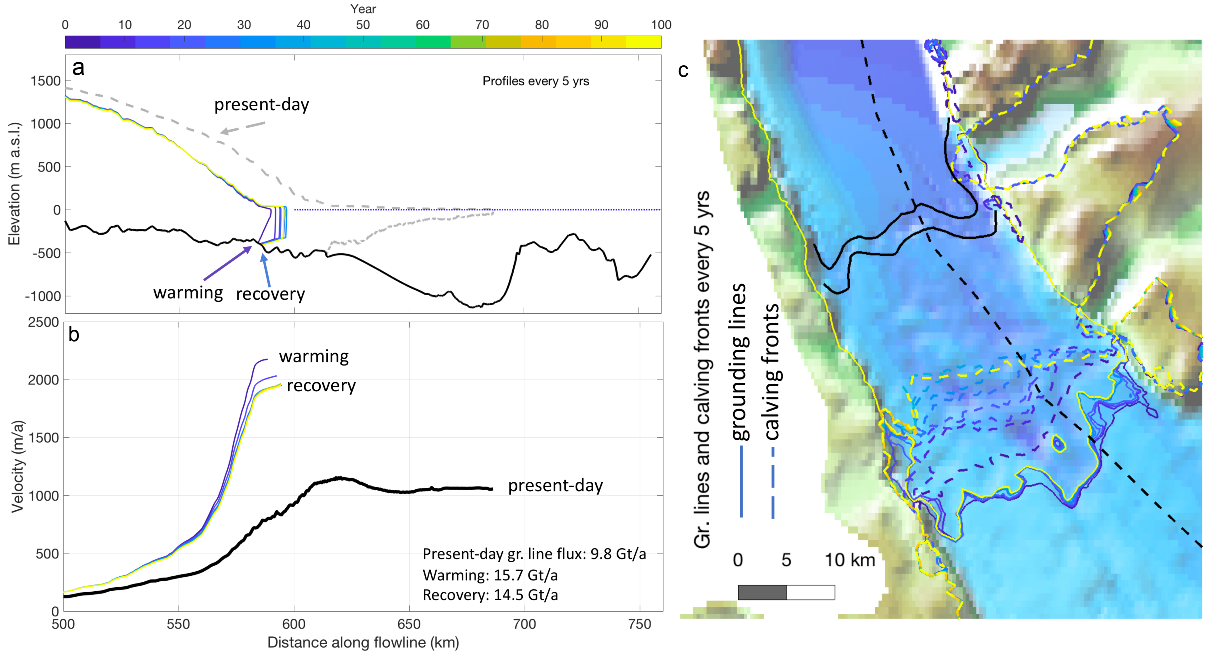

Figure S 4: (a) Geometry and (b) velocity evolution of Petermann Glacier during reversal of a 5°C ocean warming. (c) Grounding line and calving front evolution is also shown, along with the present-day grounding zone (solid black lines). Only the first 100 years of the simulation are shown for visibility; negligible change occurs over the rest of the simulation.

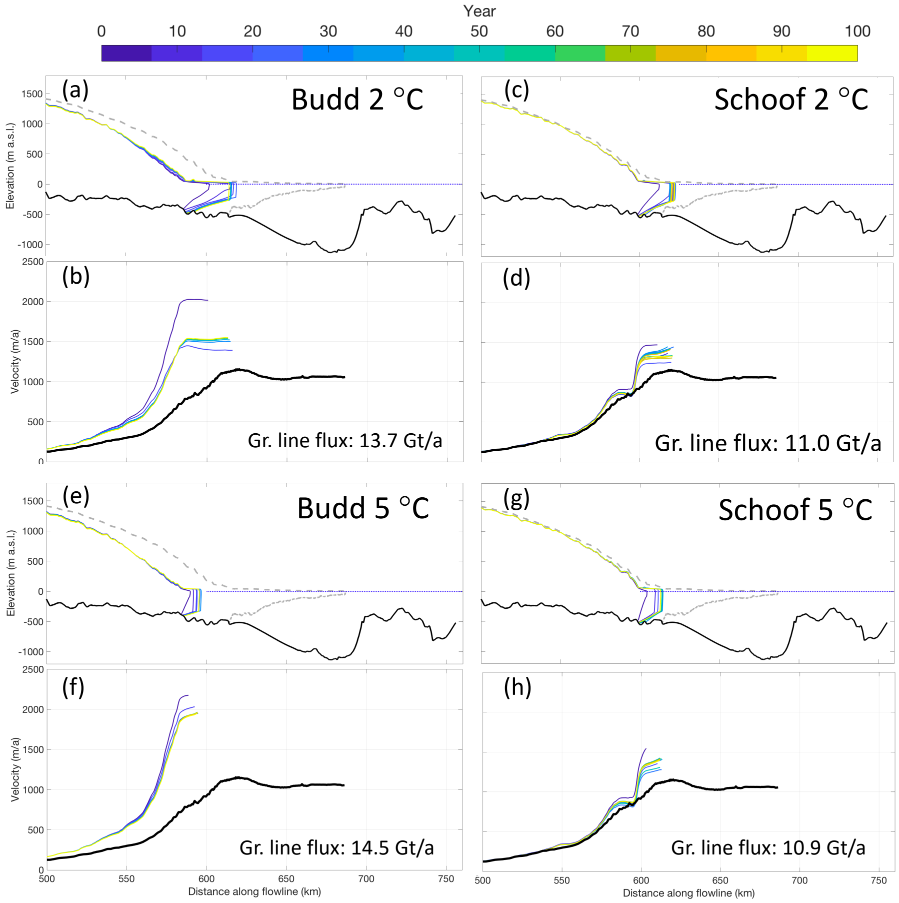

Figure S 5: Geometry and velocity evolution during recovery of Petermann Glacier from a 2 and 5 °C ocean warming, using a Budd (ab; ef) and Schoof (cd; gh) friction law. Shown are 'ocean-cooling' experiments (Section Ice-shelf breakup may cement pervasive sea-level rise), meaning no change in surface mass balance or calving parameters (see Table S1). Only the first 100 years of the simulations are shown for visibility; negligible change occurs over the rest of the simulations.

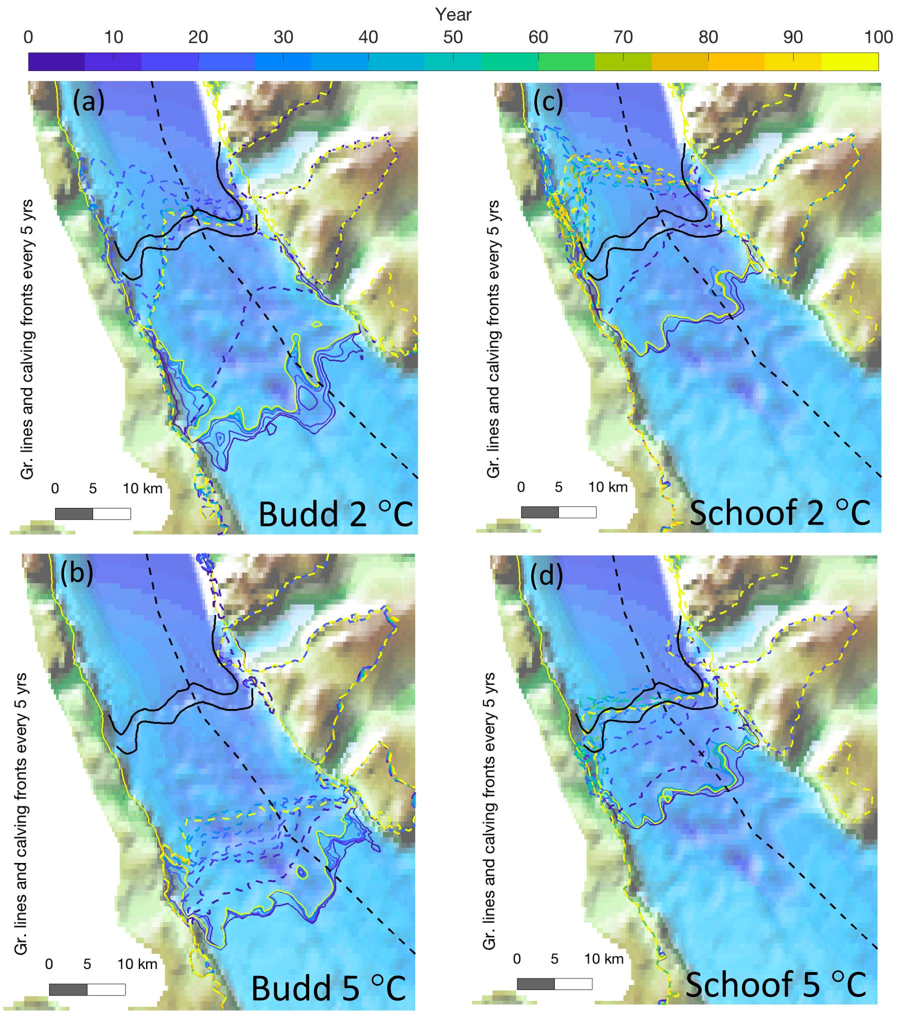

Figure S 6: Grounding line (dashed lines) and calving front (solid lines) evolution during recovery of Petermann Glacier from a 2 and 5 °C ocean warming, using a Budd (a-b) and Schoof (c-d) friction law. Shown are ‘ocean-cooling’ experiments (Section Ice-shelf breakup may cement pervasive sea-level rise), meaning no change in surface mass balance or calving parameters. Only the first 100 years of the simulations are shown for visibility; negligible change occurs over the rest of the simulations.

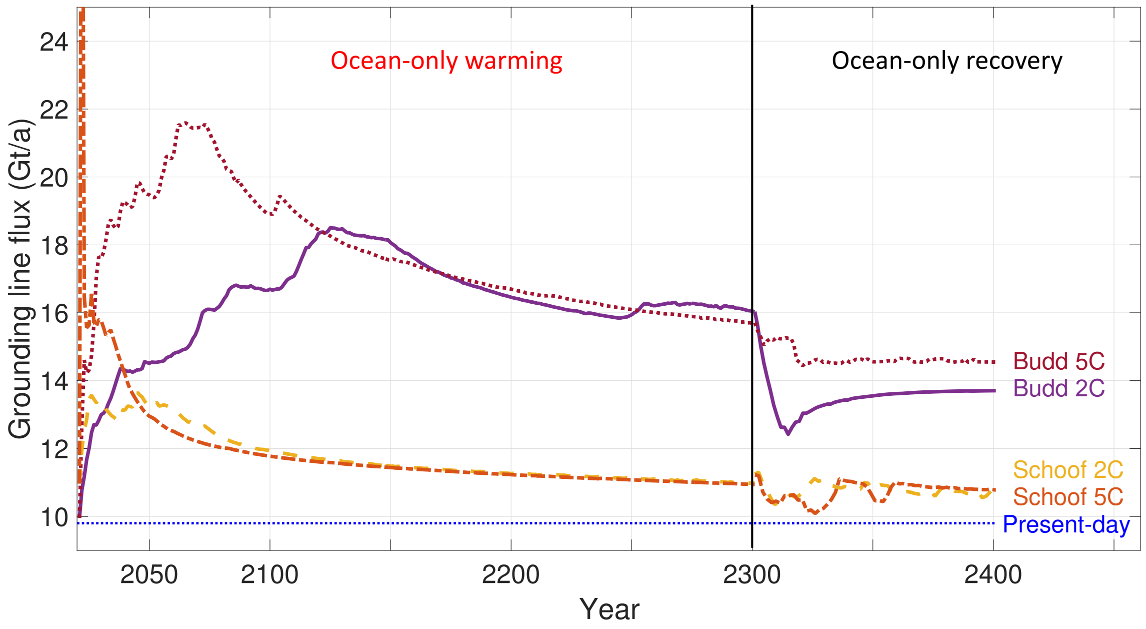

Figure S 7: Grounding-line flux evolution over time during a future ocean warming of 2 and 5 °C [5], and reversal of this warming ('ocean cooling'), using a Budd and a Schoof friction law. Shown are ocean-cooling experiments (Section Ice-shelf breakup may cement pervasive sea-level rise), meaning no change in the surface mass balance or calving parameters.

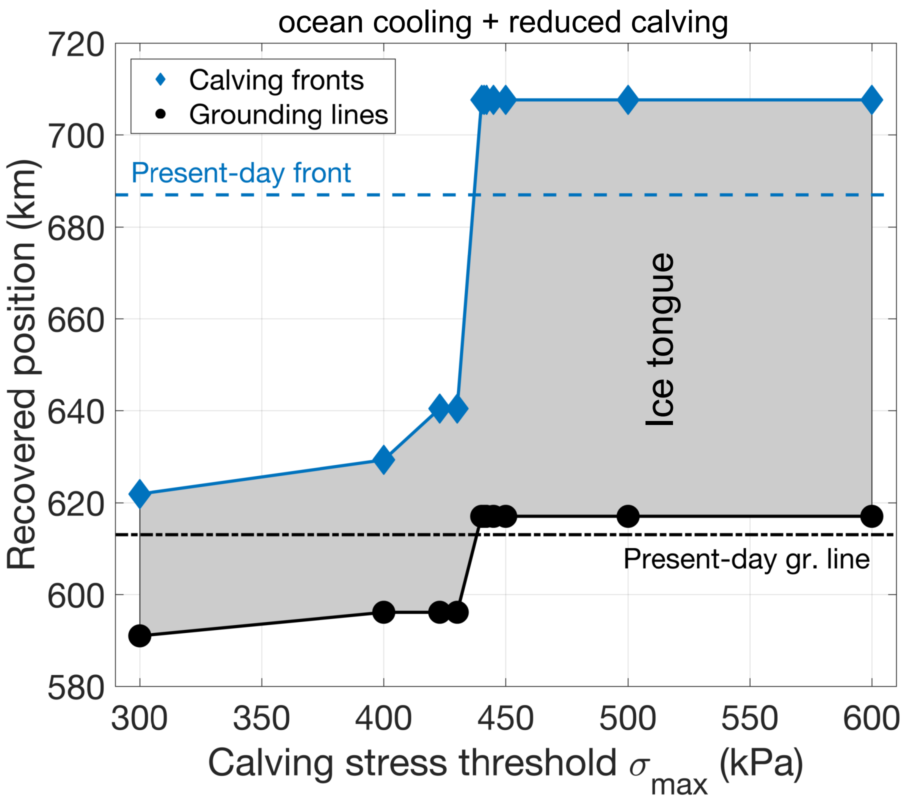

Figure S 8: Recovery under 'ocean cooling + reduced calving' depends on the imposed calving stress threshold σ_{max}. A $\sigma_{max} > 440$ kPa is required for the ice shelf to regrow to its present-day configuration. Shown are grounding-line and calving-front positions after recovery, and the ice shelf extent (gray).

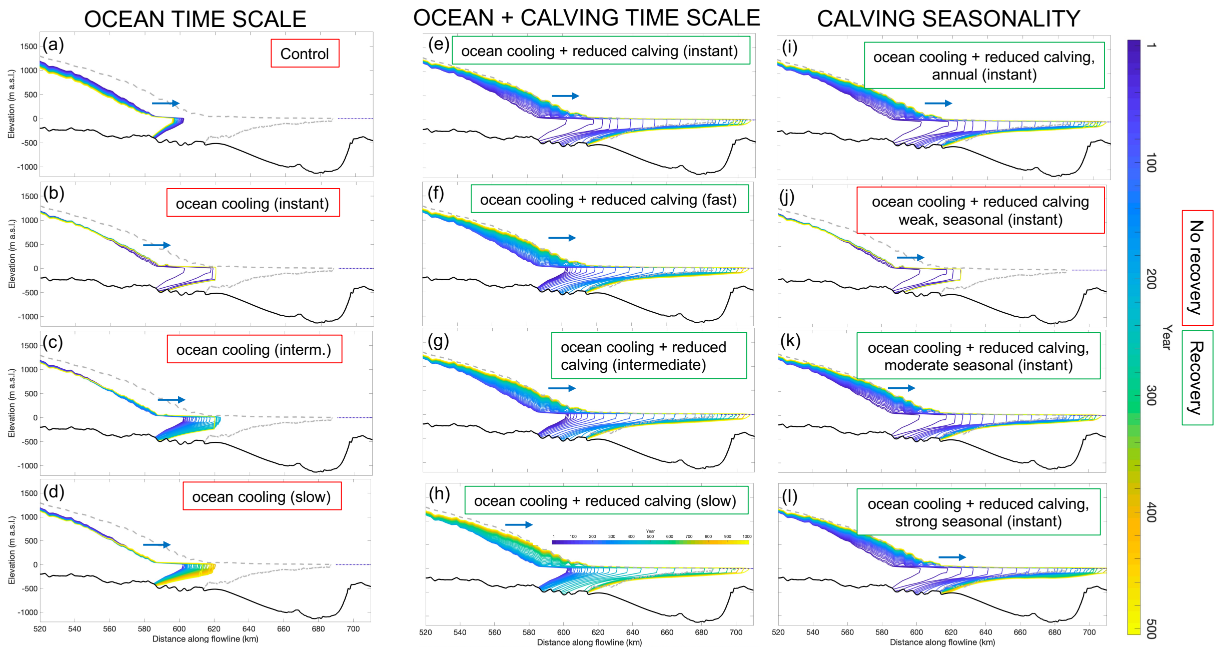

Figure S 9: Recovery of Petermann Glacier fails under an ocean-only warming reversal, no matter whether the reversal is (b) instant, (c) intermediate (over 250 years), or (d) slow (over 500 years). Analogously, recovery with 'ocean cooling + reduced calving' succeeds regardless of whether the forcing changes (e) instantly, (f) over 100 years, (g) over 250 years, or (h) over 500 years. Finally, seasonality of calving (j)–(l) needs to be strong enough (winter stress threshold $\sigma_{max} > \sim 440$ kPa in Eq. 2 in main text) to enforce ice-shelf readvance in a similar fashion as does the annual calving forcing (i). A control experiment with no change in forcing is shown in (a), meaning the future 2°C ocean warming is kept.

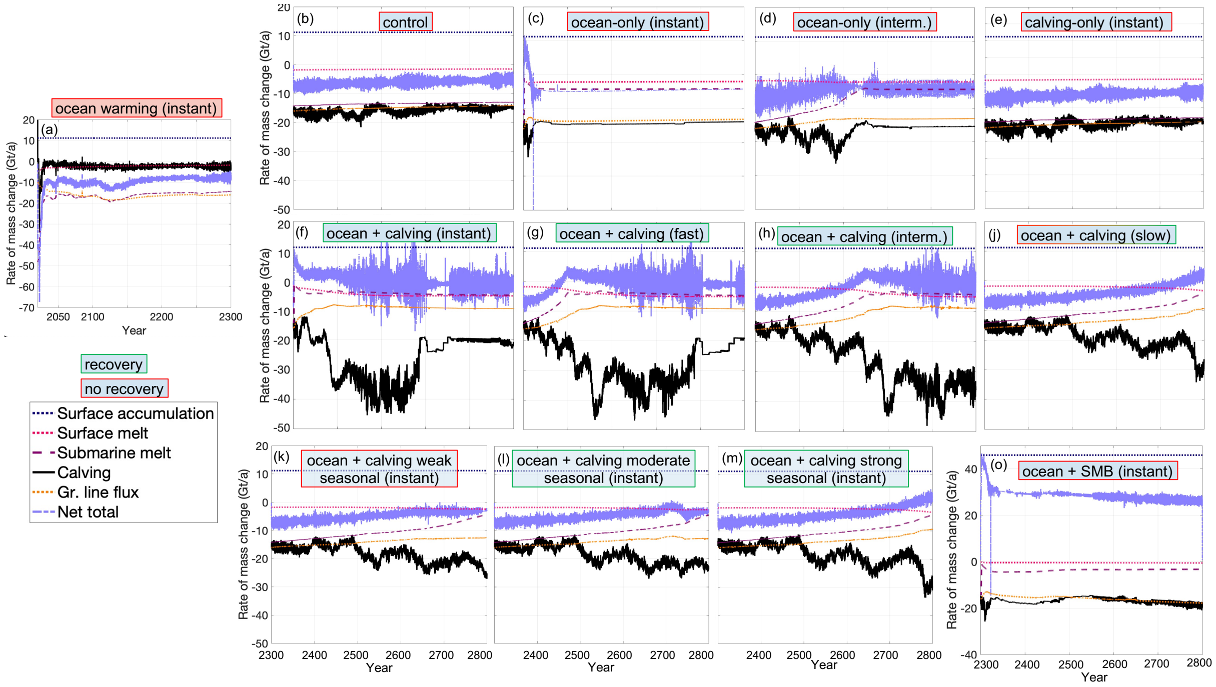

Figure S 10: Evolution of mass balance components and total volume change dV/dt ('Net total') under (a) future 2°C ocean warming [5], and (b)–(o) attempted recovery by means of various types of forcing. Grounding-line flux is shown as a negative flux for context, but is not included in the mass balance calculations (Section S1.3), as ice crossing the grounding line is not leaving the glacier system (yet). Note differing y-scale in (o). In (j) and (m), readvance is not complete after 500 years, but occurs over the subsequent centuries (not shown here). The first 500 years are shown here for time-consistency across all panels.

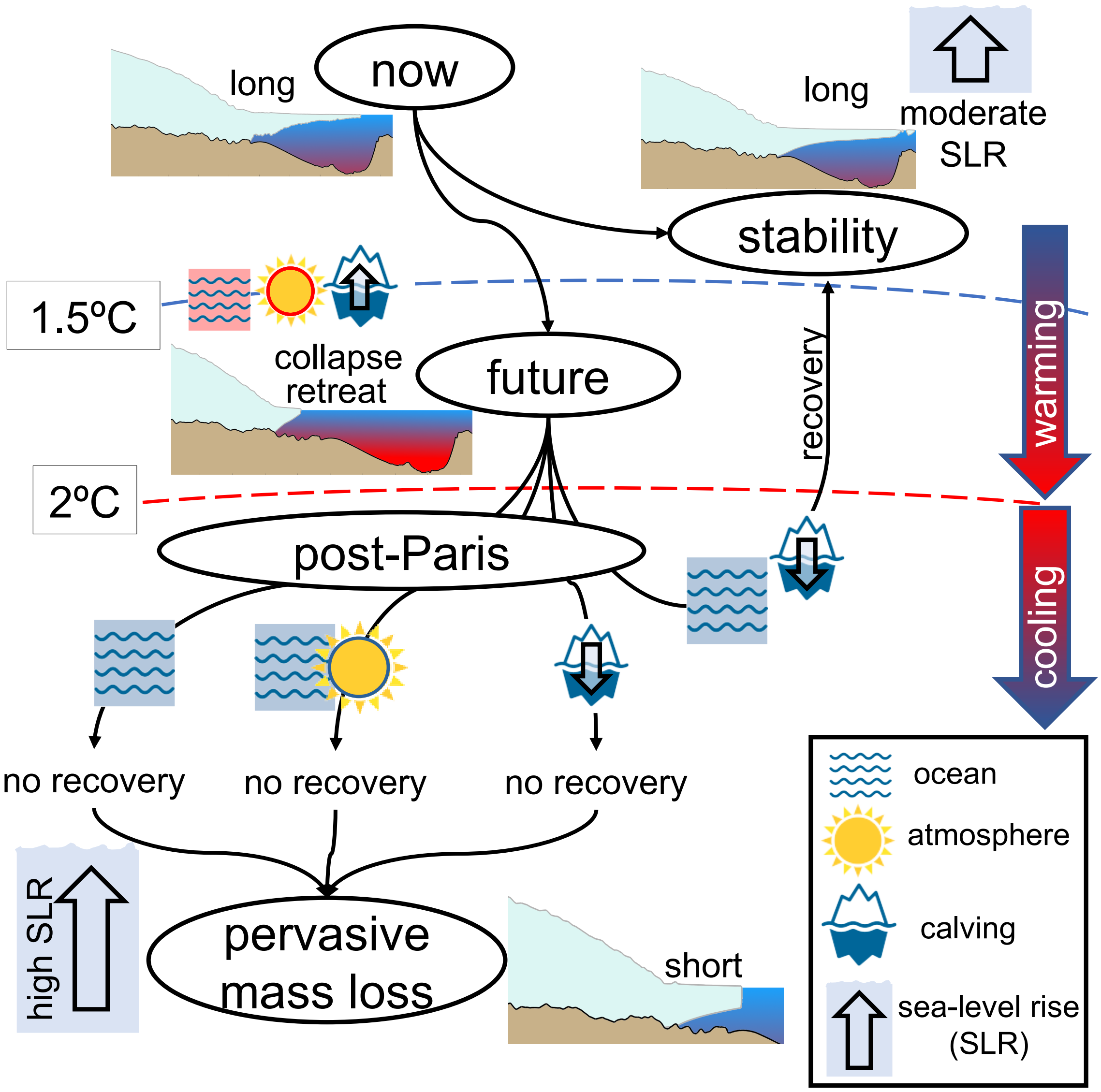

Figure S 11: Pervasive glacier-ice-shelf mass loss can be avoided if future temperature rise is kept moderate ('stability'). If carbon emissions are not reduced enough, and the goals of the Paris Agreement are not met, ice-shelf collapse and grounding-line retreat may push marine outlet glaciers into a new high-discharge state ('post-Paris'). Our model experiments suggest that it is very difficult to escape this new stable regime once ice-shelf breakup has occurred, allowing for sustained high sea-level rise ('pervasive mass loss'). A possible escape route based on our simulations is an ocean cooling coupled with sea-ice growth and thereby reduced calving rates (Section Reduced calving as an escape route from pervasive mass loss). This however likely requires a climatic cooling towards temperatures well below present-day conditions. We acknowledge the sources for the following images from Wikimedia Commons: the sun (Emoji One, CC BY-SA 4.0 https://creativecommons.org/licenses/by-sa/4.0), the ocean (BomSymbols, https://thenounproject.com/korawan_m/, CC BY 4.0), and the iceberg (Deemak Daksina, https://thenounproject.com/deemakdaksina/, CC BY 4.0 https://creativecommons.org/licenses/by/4.0).

References

[1] Johannes H Bondzio, Mathieu Morlighem, Hélène Seroussi, Michael H Wood, and Jérémie Mouginot. Control of ocean temperature on Jakobshavn Isbræ's present and future mass loss. *Geophysical Research Letters*, 45(23):12–912, 2018.

[2] Christian Schoof. The effect of cavitation on glacier sliding. *Proceedings of the Royal Society A: Mathematical, Physical and Engineering Sciences*, 461(2055):609–627, 2005.

[3] Olivier Gagliardini, D Cohen, P Råback, and Thomas Zwinger. Finite-element modeling of subglacial cavities and related friction law. *Journal of Geophysical Research: Earth Surface*, 112(F2), 2007.

[4] Julien Brondex, Fabien Gillet-Chaulet, and Olivier Gagliardini. Sensitivity of centennial mass loss projections of the Amundsen basin to the friction law. *The Cryosphere*, 13 (1):177–195, 2019.

[5] Henning Åkesson, Mathieu Morlighem, Matt O'Regan, and Martin Jakobsson. Future Projections of Petermann Glacier Under Ocean Warming Depend Strongly on Friction Law. *Journal of Geophysical Research: Earth Surface*, 126(6):e2020JF005921, 2021. doi: https://doi.org/10.1029/2020JF005921.

[6] Almut Iken. The effect of the subglacial water pressure on the sliding velocity of a glacier in an idealized numerical model. *Journal of Glaciology*, 27(97):407–421, 1981.

[7] Xavier Fettweis, Jason Box, Cécile Agosta, Charles Amory, Christoph Kittel, Charlotte Lang, Dirk van As, Horst Machguth, and Hubert Gallée. Reconstructions of the 1900–2015 Greenland ice sheet surface mass balance using the regional climate MAR model. *The Cryosphere*, 11:1015–1033, 2017.

[8] Mathieu Morlighem, Chris N Williams, Eric Rignot, Lu An, Jan Erik Arndt, Jonathan L Bamber, Ginny Catania, Nolwenn Chauché, Julian A Dowdeswell, Boris Dorschel, et al. BedMachine v3: Complete bed topography and ocean bathymetry mapping of Greenland from multibeam echo sounding combined with mass conservation. *Geophysical Research Letters*, 44(21):11–051, 2017. doi: https://doi.org/10.5067/2CIX82HUV88Y.

[9] GIMP. MEaSUREs Greenland Ice Mapping Project (GIMP) Land Ice and Ocean Classification Mask, Version 1, 2008. URL https://nsidc.org/data/NSIDC-0714/versions/1.

[10] IM Howat, A Negrete, and BE Smith. The Greenland Ice Mapping Project (GIMP) land classification and surface elevation data sets. *The Cryosphere*, 8(4):1509–1518, 2014. doi: 10.5194/tc-8-1509-2014.

[11] Ian Joughin, Ben E Smith, Ian M Howat, Ted Scambos, and Twila Moon. Greenland flow variability from ice-sheet-wide velocity mapping. *Journal of Glaciology*, 56(197): 415–430, 2010.

[12] I Joughin, B Smith, and T Scambos. MEaSUREs Multi-year Greenland Ice Sheet Velocity Mosaic, Version 1, 2016. URL https://doi.org/10.5067/QUA5Q9SVMSJG.

[13] ESA. ESA Greenland Icesheet CCI Grounding Lines from SAR Interferometry, 2017. URL http://products.esa-icesheets-cci.org/products/details/greenland_grounding_line_locations_v1_3.zip/.

[14] ESA. ESA Greenland Icesheet CCI Greenland Calving Front Locations from ERS, Sentinel-1 and LANDSAT data, 2017. URL http://products.esa-icesheets-cci.org/products/details/greenland_calving_front_locations_v3_0.zip/.

[15] Cilan Cai, Eric Rignot, Dimitris Menemenlis, and Yoshihiro Nakayama. Observations and modeling of ocean-induced melt beneath Petermann Glacier Ice Shelf in northwestern Greenland. *Geophysical Research Letters*, 44(16):8396–8403, 2017.

[16] GIMP. MEaSUREs Greenland Ice Mapping Project (GIMP) Digital Elevation Model, Version 1, 2010. URL https://nsidc.org/data/nsidc-0645/versions/1.
